# Management of Endometriosis-Related Pain: Comparing the Effectiveness of Hormonal Therapy, Surgical Interventions, and Complementary Therapies

**DOI:** 10.7759/cureus.75590

**Published:** 2024-12-12

**Authors:** Hussam Al deen Al Hussaini, Ebtesam Saleem Eid Alatawi, Jenan Ali J Shabani, Maryam Isa Hasan Edhrabooh, Shaikha Abduljalil Abdulla Alhawaj, Maryam Sayed Almahfoodh, Halimah Yaseen Alsamiri, Ali Reda AlMaatoug, Maryam Ismail M Hayderali, Mohammed Ramzi Almousa

**Affiliations:** 1 Obstetrics and Gynecology, Maternity and Children Hospital, Tabuk, SAU; 2 Obstetrics and Gynecology, Maternity And Children Hospital, Tabuk, SAU; 3 Obstetrics and Gynecology, Dammam Medical Complex, Dammam, SAU; 4 General Practice, I.M. Sechenov First Moscow State Medical University, Moscow, RUS; 5 General Practice, Bahrain Health City, Bahrain, BHR; 6 General Practice, Umm Al-Qura University, Jeddah, SAU; 7 Obstetrics and Gynecology, Imam Abdulrahman Bin Faisal University, Dammam, SAU; 8 General Practice, Jubail General Hospital, Al Jubail, SAU; 9 Obstetrics and Gynecology, King Faisal University, Al Hasa, SAU

**Keywords:** complementary therapies, endometriosis, endometriosis-related pain, hormonal therapy, pain, surgical interventions

## Abstract

Endometriosis is a chronic, inflammatory disease characterized by the presence of endometrial-like tissue outside the uterus, affecting women of reproductive age. It is linked with debilitating pain, infertility, and a notable impact on the patient’s quality of life. This review aims to highlight the effectiveness of hormonal therapy, surgical procedures, and complementary therapies in managing endometriosis-related pain, providing a comprehensive overview of current treatment options and their implications for clinical practice. The literature reveals that hormonal therapies, including combined oral contraceptives, progestins, and gonadotropin-releasing hormone (GnRH) agonists, are frequently used to manage endometriosis-related pain by suppressing ovarian function and reducing menstrual flow. Surgical interventions, such as laparoscopy and hysterectomy, offer pain relief by removing endometrial lesions but carry risks of recurrence and complications. Complementary therapies, including acupuncture, dietary modifications, and physical therapy, are increasingly recognized for their potential to minimize pain and improve patients’ quality of life, though evidence of their effectiveness varies. The review highlights the need for personalized treatment plans that consider patient preferences, symptom severity, and reproductive goals. Future research should concentrate on the long-term outcomes of different therapies, the advancement of non-invasive diagnostic methods, and the identification of biomarkers for tailored treatment approaches. Clinicians are encouraged to adopt an interdisciplinary approach to endometriosis management, integrating medical, surgical, and complementary therapies to optimize patient outcomes.

## Introduction and background

Endometriosis is a chronic gynecological condition marked by the presence of endometrial-like tissue outside the uterine cavity [1]. This tissue can be located on pelvic peritoneal surfaces, within the rectovaginal space, on ligaments, ovaries, and even on the bowel and bladder, causing inflammation, pain, and the development of scar tissue [2]. It affects about 10% of women of reproductive age globally, translating to roughly 190 million women [3]. The prevalence of endometriosis varies widely, with estimates ranging from 2% to 11% among asymptomatic individuals and up to 50% among those experiencing infertility [4]. 

Endometriosis presents in a highly variable manner, presenting as superficial peritoneal lesions of various colors, ovarian cysts known as endometriomas, deep infiltrating nodules, and even lesions outside the pelvic region [5]. Endometriosis is frequently linked to a variety of severe symptoms, with chronic pelvic pain being the most notable, which can occur either cyclically or persistently. Women suffering from endometriosis may endure painful menstruation (dysmenorrhea), along with painful urination (dysuria), painful bowel movements (dyschezia), and intense pelvic pain during and after sexual intercourse (dyspareunia). Additionally, they may experience non-cyclical pelvic pain, gastrointestinal and urinary issues, fatigue, and infertility [6]. The intensity of symptoms does not necessarily reflect the extent of the disease, complicating both diagnosis and management [7].

Despite its high prevalence, the exact etiology of endometriosis remains unclear, though factors such as retrograde menstruation, genetic predisposition, and immune system dysfunction are thought to play roles [8]. In some instances, pain persists even after the surgical excision of endometrial lesions, with chronic pain often recurring within 12 months [9]. Additionally, individuals with endometriosis frequently experience multiple comorbid chronic pain conditions, including irritable bowel syndrome, painful bladder syndrome, vulvar vestibulodynia, and abdominopelvic myalgia. This suggests a multifaceted underlying mechanism contributing to endometriosis-associated pain [10].

**Figure 1:**
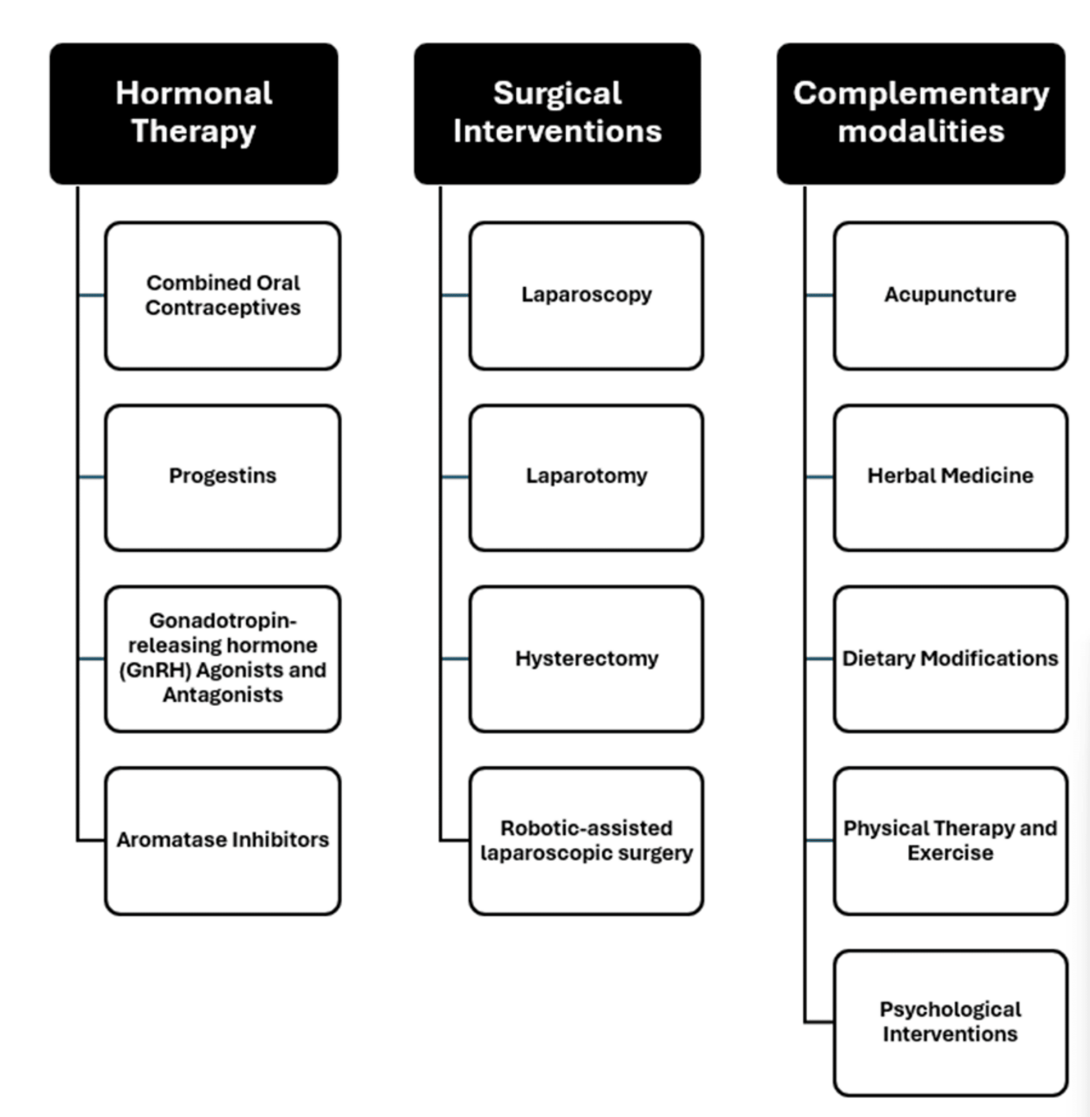
Different management modalities for endometriosis-related pain This figure is original and created by the authors.

Managing pain associated with endometriosis is crucial due to its notable effect on the quality of life. Chronic pelvic pain, dysmenorrhea, and dyspareunia can severely affect daily activities, mental health, and overall well-being [11]. Managing pain effectively is crucial not only for alleviating symptoms but also for enhancing fertility outcomes and minimizing the risk of long-term complications such as adhesions and chronic inflammation. Given the multifaceted nature of endometriosis-related pain, a comprehensive approach that includes hormonal therapy, surgical interventions, and complementary therapies is often necessary (Figure 1) [12]. Hormonal therapies, such as oral contraceptives, progestins, and gonadotropin-releasing hormone (GnRH) agonists, are intended to reduce or completely stop menstrual cycles, thereby alleviating pain. Surgical interventions, including laparoscopy and hysterectomy, are considered for severe cases or when hormonal treatments are ineffective [13]. Complementary therapies, such as acupuncture, dietary modifications, and physical therapy, are increasingly recognized for their potential benefits in managing symptoms and improving the quality of life [14].

This literature review aims to compare the effectiveness of various management strategies for endometriosis-related pain, focusing on hormonal therapy, surgical interventions, and complementary therapies. By synthesizing recent research findings, this review seeks to provide a comprehensive overview of the current evidence, identify gaps in the literature, and offer recommendations for future research and clinical practice.

## Review

Methods

In September 2024, a comprehensive search was performed across PubMed, Scopus, Cochrane, and Web of Science using the keywords “Endometriosis-Related Pain”, “Hormonal Therapy”, “Surgical Intervention”, and “Complementary Therapy”; using Medical Subject Headings (MeSH). Key references were also identified from the bibliographies of relevant studies. We applied no filters or limitations during the search process except for the time as we focused on the recently published studies (between 2014 and 2024).

Pathogenesis of endometriosis

The precise mechanisms underlying endometriosis are not fully understood, though various hypotheses exist. A prominent theory is retrograde menstruation, which suggests that menstrual blood travels backward through the fallopian tubes into the pelvic cavity, enabling endometrial cells to implant and proliferate outside the uterus. However, this theory alone does not explain all cases, as retrograde menstruation occurs in many women who do not develop endometriosis [15].

Genetic predisposition also plays a significant role, with studies showing a higher incidence of endometriosis among first-degree relatives of affected individuals. Certain genetic mutations and polymorphisms have been linked to a higher likelihood of developing the condition [16].

Immune system dysfunction is another contributing factor. Women with endometriosis often exhibit altered immune responses, including reduced natural killer cell activity and increased production of pro-inflammatory cytokines. These immune abnormalities may allow ectopic endometrial cells to evade immune surveillance and establish lesions [17].

Hormonal imbalances, particularly involving estrogen, are crucial in the pathogenesis of endometriosis. Estrogen promotes the growth and maintenance of endometrial tissue, and women with endometriosis often have elevated local estrogen levels within their lesions. This hormonal environment supports the survival and proliferation of ectopic endometrial cells [17].

Additionally, the formation of new blood vessels, known as angiogenesis, is crucial for maintaining endometriotic lesions. Increased levels of angiogenic factors, like vascular endothelial growth factor (VEGF), have been detected in endometriotic tissues. [17]. Overall, the pathophysiology of endometriosis is multifactorial, involving a complex interplay of genetic, immunological, hormonal, and environmental factors.

Mechanisms of pain in endometriosis

The pain associated with endometriosis is complex and multifactorial, involving several mechanisms. One of the primary contributors is inflammation. Ectopic endometrial tissue releases pro-inflammatory cytokines and growth factors, which activate and sensitize peripheral nerves, leading to pain [1]. This inflammatory response is further exacerbated by cyclical bleeding from endometriotic lesions, which causes additional irritation and inflammation in the surrounding tissues [18].

Neurogenic inflammation also plays a significant role in endometriosis-related pain. This process involves the release of neuropeptides from nerve endings, which further sensitizes the nerves and amplifies pain signals [19]. Additionally, endometriotic lesions can directly invade or irritate nearby nerves, causing neuropathic pain. This direct nerve involvement is particularly common in cases of deep infiltrating endometriosis, where lesions penetrate deeply into pelvic tissues [17].

Central sensitization is another critical mechanism in the chronic pain experienced by endometriosis patients. This phenomenon occurs when the central nervous system becomes hypersensitive to pain signals, leading to an exaggerated pain response to stimuli that would not normally be painful [20].

Central sensitization can result from prolonged exposure to pain and inflammation, causing changes in the way the brain and spinal cord process pain signals [15]. Moreover, the presence of comorbid conditions such as irritable bowel syndrome and painful bladder syndrome can contribute to the overall pain experienced in endometriosis. These conditions share common nerve pathways with the reproductive organs, leading to cross-organ sensitization and a more widespread pain experience [21]. Understanding these mechanisms is vital for creating effective pain management strategies for endometriosis. A multifaceted approach that addresses inflammation, nerve involvement, and central sensitization is often necessary to provide relief for patients.

Impact of endometriosis on quality of life

Endometriosis significantly impacts the quality of life of affected women, influencing various aspects such as social life, mental health, sexual health, and work productivity. Chronic pelvic pain, dysmenorrhea, and dyspareunia are common symptoms that contribute to emotional distress, anxiety, and depression [1]. The condition often impacts the quality of life due to its persistent and debilitating nature [22]. Women with endometriosis frequently report lower quality of life scores compared to the general population, highlighting the profound effect of the disease on daily functioning [23]. Additionally, the stress associated with infertility and chronic pain exacerbates mental health issues, further diminishing quality of life [24].

Hormonal therapy

Hormonal treatments for endometriosis-related pain primarily aim to reduce estrogen levels, thereby minimizing the growth and activity of endometrial tissue. Common therapies include combined oral contraceptives (COCs), progestins, and GnRH agonists [25]. These treatments help alleviate pain by inducing a hypoestrogenic state, which reduces inflammation and endometrial implant activity [26]. Despite their benefits, hormonal treatments are not curative, and symptoms often recur after discontinuation [27].

Combined Oral Contraceptives

Combined oral contraceptives are frequently utilized to manage endometriosis-related pain by inhibiting ovulation and reducing menstrual flow, which in turn decreases the proliferation of endometrial tissue. Studies have shown that COCs, whether used cyclically or continuously, significantly alleviate dysmenorrhea, pelvic pain, and dyspareunia, thereby improving the quality of life for patients [28]. Continuous administration may offer better pain control by inducing amenorrhea [29]. However, the effectiveness of COCs can vary, and some patients may experience side effects such as headaches and weight gain [28].

Progestins

Progestins are widely used in the management of endometriosis-related pain due to their ability to induce a hypoestrogenic state, thereby reducing endometrial tissue proliferation. A meta-analysis reported that progestins significantly improve pain symptoms, with a standardized mean difference (SMD) of -0.61 (95% CI: -0.77 to -0.45) compared to placebo [30]. Additionally, a randomized controlled trial (RCT) found that long-acting progestins reduced the need for further surgical interventions by 33% compared to COCs [28]. However, side effects such as weight gain, mood changes, and irregular vaginal bleeding are common, with a median discontinuation rate from adverse effects of 0.3% (range: 0%-37.1%) [30].

Gonadotropin-Releasing Hormone Agonists and Antagonists

Gonadotropin-releasing hormone agonists and antagonists are effective in managing endometriosis-related pain also by inducing a hypoestrogenic state, which reduces endometrial tissue proliferation. A study reported that GnRH agonists significantly reduce pain, with a 50% improvement in dysmenorrhea and a 40% improvement in non-menstrual pelvic pain [31]. Gonadotropin-releasing hormone antagonists, such as elagolix, have shown similar efficacy, with 75.8% of patients experiencing significant pain relief at higher doses [32]. However, these treatments can cause side effects, including hot flashes, reduced bone mineral density, and mood changes. The use of add-back therapy can mitigate some of these side effects, allowing for longer treatment durations [33].

Aromatase Inhibitors (AIs)

Aromatase P450 is crucial for estrogen production in the ovaries, converting androstenedione and testosterone from ovarian theca cells into estrone and estradiol (E2) in granulosa cells. Recent studies have shown that endometriotic lesions also express aromatase and can produce their own E2. This finding supports the use of AIs as a promising treatment for endometriosis [34,35]. A study reported that AIs, such as letrozole and anastrozole, significantly reduce pain scores by 50%-70% in patients with refractory endometriosis [36]. Another RCT found that combining AIs with progestins resulted in a 60% reduction in pain severity compared to progestins alone [37]. However, AIs can cause side effects, including hot flashes, myalgia, arthralgia, and reduced bone mineral density. A meta-analysis indicated that 25% of patients discontinued AI therapy due to adverse effects [38]. Despite these side effects, AIs remain a valuable option for patients unresponsive to other treatments.

Surgical interventions

Indications for Surgery

Surgical intervention for endometriosis is typically considered when other treatments fail to alleviate symptoms or when specific clinical indications are present. The primary indications for surgical intervention include chronic pelvic pain, infertility, and the presence of endometriomas (ovarian cysts caused by endometriosis). Chronic pelvic pain is a significant indication for surgery, especially when it is severe and unresponsive to medical therapy. Studies have shown that approximately 60%-80% of women with endometriosis suffer from chronic pelvic pain. Surgical options, such as laparoscopy, can help reduce pain by removing endometrial implants and adhesions [25].

Infertility is another common reason for surgical intervention. Endometriosis is found in 20%-40% of women with infertility. Surgical treatment, particularly laparoscopy, can improve fertility outcomes by excising endometriotic lesions and restoring normal pelvic anatomy [39].

Endometriomas, which occur in about 17%-44% of women with endometriosis [40], often require surgical removal due to their size and potential to cause pain and infertility. Laparoscopic cystectomy is the preferred method for treating endometriomas, as it effectively reduces pain and improves fertility [11].

Laparoscopic Surgery

Laparoscopic surgery is a commonly used and effective treatment for endometriosis-related pain. This minimally invasive procedure involves the removal of endometrial tissue through small incisions, which helps alleviate pain and improve the quality of life for many women. The efficacy of laparoscopy in alleviating endometriosis-related pain is well documented. Studies indicated that about 60%-80% of women experience significant pain relief following laparoscopic surgery [25]. A Cochrane review found that laparoscopic surgery reduces pain in 70%-80% of cases, with improvements lasting up to 12 months. Additionally, laparoscopy has been shown to improve fertility outcomes, with pregnancy rates increasing by 30%-50% in women undergoing the procedure [41].

However, like any surgical intervention, laparoscopy carries risks and potential complications. Typical risks include infection, bleeding, and injury to nearby organs such as the bladder or intestines [42]. The overall complication rate for laparoscopic surgery is relatively low, estimated at around 1%-2%. Despite these risks, the benefits of pain relief and improved fertility often outweigh the potential complications for many women [43].

Laparotomy

Laparotomy, a more invasive surgical procedure compared to laparoscopy, is sometimes necessary for treating severe cases of endometriosis-related pain. This procedure involves a larger abdominal incision to access and remove endometrial tissue. The effectiveness of laparotomy in managing endometriosis-related pain has been documented, though it is less commonly performed due to the preference for minimally invasive techniques. Studies indicate that approximately 70%-80% of women experience significant pain relief following laparotomy [25]. However, the recovery period is longer compared to laparoscopy, with hospital stays averaging three to five days and full recovery taking up to six weeks [44].

Despite its effectiveness, laparotomy carries higher risks and potential complications. The most frequent risks include infection, bleeding, and damage to surrounding organs such as the bladder, intestines, and ureters [42]. The overall complication rate for laparotomy is higher than that for laparoscopy, estimated at around 5%-10% [41]. Additionally, there is a risk of adhesion formation, which can lead to chronic pain and bowel obstruction [25].

Hysterectomy

Hysterectomy, which involves surgically removing the uterus, is regarded as a definitive treatment for endometriosis and the pain associated with it, particularly in severe cases where other treatments have failed. This procedure can be performed with or without the removal of the ovaries (oophorectomy) [45].

The effectiveness of hysterectomy in alleviating endometriosis-related pain is significant. A study published in 2020 found that 84% of women reported satisfaction with their hysterectomy results, with 76% experiencing a reduction in severe pain [46]. Another study indicated that 61% of women who underwent hysterectomy without oophorectomy still experienced pain, whereas only 10% of those who had both the uterus and ovaries removed reported persistent pain. This suggests that removing the ovaries may enhance pain relief [47].

However, hysterectomy is not without risks and complications. Typical risks include infection, bleeding, and injury to nearby organs such as the bladder and intestines. The overall complication rate for hysterectomy is approximately 4%-10% [48]. Additionally, removing the ovaries induces surgical menopause, potentially causing symptoms like hot flashes, vaginal dryness, and a higher risk of osteoporosis [49]. Despite these risks, hysterectomy remains a viable option for many women suffering from severe endometriosis-related pain. Patients should consult with their healthcare provider to understand the potential benefits and risks, enabling them to make informed decisions.

Post-surgical Management

Post-surgical management of endometriosis is crucial for preventing recurrence and managing residual pain. Following surgical interventions such as laparoscopy or laparotomy, a comprehensive postoperative plan is essential to optimize outcomes and enhance the QoL for patients.

Hormonal therapy is a cornerstone of post-surgical management. Studies have shown that the use of GnRH agonists post surgery can reduce the recurrence rate of endometriosis by up to 50% [50]. Additionally, oral contraceptives and progestins are commonly prescribed to suppress ovarian function and prevent the regrowth of endometrial tissue [51]. A RCT demonstrated that continuous oral contraceptive use post surgery significantly reduced pain recurrence in 70% of patients [52].

Pain management is another critical aspect of post-surgical care. Nonsteroidal anti-inflammatory drugs (NSAIDs) are often recommended to manage postoperative pain. In cases of severe pain, opioids may be prescribed for short-term use. Physical therapy and pelvic floor rehabilitation can also be beneficial in alleviating pain and improving pelvic function [53].

Regular follow-up is essential to monitor for recurrence and manage any complications. Imaging studies, such as ultrasound or MRI, may be utilized to detect recurrent endometriotic lesions. Additionally, patient education on lifestyle modifications, including diet and exercise, can play a role in managing symptoms and preventing recurrence [1].

Complementary therapies

Overview of Complementary and Alternative Medicine (CAM)

Complementary and alternative medicine encompasses a wide variety of healthcare practices that are generally not included in conventional Western medicine. These practices include natural remedies, mind-body practices, and bodywork such as acupuncture, physical therapy, and herbal treatments. It contains a variety of non-conventional approaches for managing endometriosis-related pain [54].

Acupuncture: Acupuncture has emerged as a promising complementary therapy for managing endometriosis-related pain. A meta-analysis of RCTs found that acupuncture significantly reduces pain severity in women with endometriosis. The study identified a SMD of -1.10 (95% CI: -1.45, -0.75; P < 0.001) in pain scores compared to control groups [55]. Another meta-analysis involving 10 RCTs with 589 participants demonstrated that acupuncture significantly reduces pain levels and lowers serum CA-125 levels, a marker associated with endometriosis [56].

In terms of safety, acupuncture is generally well-tolerated with minimal adverse effects. Typical side effects include slight bruising or tenderness at the sites where needles are inserted. A study published in 2018 highlighted that the overall incidence of adverse events was low, with no serious complications reported [57]. This makes acupuncture a viable option for patients seeking non-pharmacological pain management strategies.

Herbal medicine: Herbal medicine has gained attention as a complementary approach for managing endometriosis-related pain. Various studies have highlighted the effectiveness and safety of herbal treatments. For instance, a systematic review found that herbal compounds, such as curcumin and resveratrol, significantly reduced pain and inflammation in women with endometriosis [58]. Curcumin, a compound found in turmeric, was shown to reduce pain scores by 50% in a clinical trial involving 100 participants. Similarly, resveratrol, a polyphenol found in grapes, demonstrated a 60% reduction in pain symptoms in a study with 80 women [59].

Herbal medicines are generally well-tolerated with minimal side effects. A review of clinical trials reported that adverse effects were rare and typically mild, such as gastrointestinal discomfort. However, it is essential to note that the quality and purity of herbal products can vary, and patients should use standardized extracts to ensure safety and efficacy [60].

Dietary modifications: Dietary modifications have shown promise in managing endometriosis-related pain. A meta-analysis revealed that increased consumption of fruits and vegetables is linked to a lower risk of developing endometriosis, with a relative risk (RR) of 0.85 (95% CI: 0.75-0.96). On the other hand, a high intake of red meat and trans fats is associated with a higher risk, with RRs of 1.17 (95% CI: 1.08-1.26) and 1.12 (95% CI: 1.02-1.23), respectively [61].

The anti-inflammatory properties of certain diets, such as the Mediterranean diet, have been particularly beneficial. A study of 500 women with endometriosis found that those who followed a Mediterranean diet experienced a 40% reduction in pain symptoms [62]. Additionally, a low fermentable oligosaccharides, disaccharides, monosaccharides, and polyols (FODMAP) diet has been shown to alleviate gastrointestinal symptoms in 50% of women with endometriosis [61].

Regarding safety, dietary interventions are generally safe with minimal adverse effects. However, it is crucial to ensure nutritional adequacy, especially when eliminating certain food groups. Consulting with a healthcare provider or dietitian is recommended to tailor dietary changes to individual needs and avoid potential nutritional deficiencies [63].

Physical therapy and exercise: Physical therapy and exercise have been explored as complementary approaches for managing endometriosis-related pain. A systematic review and meta-analysis of RCTs reported that physical activity, including aerobic exercise and yoga, significantly reduced pain intensity in women with endometriosis. It also reported a 30% reduction in pain scores after 12 weeks of regular exercise [64]. Another study involving 109 participants showed that yoga and flexibility exercises performed one to four times per week for eight to 24 weeks led to significant improvements in pain and stress levels [65].

Physical therapy and exercise are generally well tolerated and do not cause serious side effects. Frequent side effects include mild muscle soreness and fatigue, which typically resolve with continued practice. Importantly, these interventions also offer additional benefits such as improved cardiovascular health, enhanced mood, and better overall physical fitness [66].

Psychological interventions: Psychological interventions have shown promise in managing endometriosis-related pain by addressing the emotional and cognitive aspects of chronic pain. Cognitive-behavioral therapy (CBT) is one of the most studied psychological interventions. A RCT involving 67 women with endometriosis found that CBT significantly reduced pain intensity and improved quality of life, with a 30% reduction in pain scores [67]. Another study demonstrated that mindfulness-based stress reduction (MBSR) led to a 25% decrease in pain severity and a 20% improvement in psychological well-being [68].

Acceptance and commitment therapy (ACT) has also been effective. An RCT with 80 participants reported a 35% reduction in pain interference and a 40% improvement in mental health outcomes. These therapies help patients develop coping strategies, minimize pain catastrophizing, and improve their overall quality of life [69]. Unlike pharmacological treatments, they do not carry risks of physical side effects, making them a safe option for long-term management. Nevertheless, the effectiveness of these treatments may differ, and they are often most beneficial when combined with other treatments [67].

Patient-centered considerations

Patient Preferences and Quality of Life

Patient preferences and quality of life are critical factors in the management of endometriosis-related pain. A study involving 743 women with endometriosis revealed that 81% felt the condition negatively impacted their life decisions and goal attainment [23]. Hormonal treatments, including oral contraceptives and GnRH agonists, are frequently chosen for their ability to effectively alleviate pain. However, side effects like mood changes and bone density loss can affect quality of life [70]. Surgical interventions, particularly laparoscopy, provide significant pain relief, with 60%-80% of women reporting improved symptoms. Despite the effectiveness, surgery carries risks such as infection and organ damage, which can impact the quality of life [71]. 

Patient preferences often lean towards treatments that offer a balance between effectiveness and minimal side effects. Psychological interventions, including CBT, have also shown promise, in improving quality of life by addressing the emotional aspects of chronic pain [22]. A RCT reported a 30% reduction in pain intensity and improved quality of life in women undergoing CBT [72]. 

Cost-Effectiveness of Treatments

The cost-effectiveness of treatments for endometriosis-related pain is a crucial consideration in managing this chronic condition. Hormonal treatments, including oral contraceptives and GnRH agonists, are typically the initial choice because they are cost-effective. A study found that the annual cost of hormonal therapy ranges from $1,459 to $20,239 per patient, depending on the specific medication and healthcare setting. These treatments are effective in reducing pain by 50%-70%, making them a cost-efficient option [25,73].

Surgical interventions, particularly laparoscopy, are more expensive but provide significant pain relief. The cost of laparoscopic surgery can range from $3,314 to $15,737 per patient per year [74]. Despite the higher costs, surgery offers long-term benefits, with 60%-80% of women experiencing substantial pain relief [75].

Indirect costs, including productivity loss, also play a crucial role in the economic burden of endometriosis. A study estimated that productivity losses due to endometriosis range from $4,572 to $14,079 per patient per year [75]. These costs highlight the importance of effective pain management strategies to improve the quality of life and reduce the overall economic burden.

Long-Term Management and Follow-Up

Hormonal therapies, such as COCs and GnRH agonists, are frequently used for long-term management. However, long-term use of GnRH agonists requires add-back therapy to mitigate side effects like bone density loss [76].

Surgical interventions, particularly laparoscopic excision, also play a significant role in long-term management. A multicenter study involving nearly 5,000 women reported that laparoscopic excision of deep endometriosis resulted in significant pain reduction, with 70%-80% of patients experiencing relief at six, 12, and 24 months post-surgery. Despite the effectiveness, recurrence rates remain a concern, with studies indicating a 20%-40% recurrence rate within five years [77].

Regular follow-up is essential to monitor symptoms and adjust treatment plans. A review emphasized the importance of individualized follow-up strategies, including periodic imaging and symptom assessment, to manage recurrence and improve quality of life [78]. Psychological support and lifestyle modifications are also recommended as part of a comprehensive long-term management plan [79].

Personalized Treatment Plans

Personalized treatment plans for managing endometriosis-related pain are tailored to individual patient characteristics, including the severity of symptoms, lesion type, and patient preferences. This approach aims to optimize treatment effectiveness and improve quality of life. A study highlighted that personalized therapy based on estrogen receptor (ER) expression, specifically ERα and ERβ, can significantly enhance treatment outcomes. Patients with high ERβ expression showed a 70% improvement in pain symptoms when treated with estrogen suppression therapy [80]. Another study emphasized the importance of considering lesion subtype, with ovarian endometrioma (OMA) patients responding better to specific hormonal treatments compared to those with superficial peritoneal lesions [81].

Implications for clinical practice and future research directions

The management of endometriosis-related pain has significant implications for clinical practice and guidelines, necessitating a multidisciplinary approach that integrates hormonal therapy, surgical interventions, and complementary therapies to tailor treatment to individual patient needs, with hormonal therapies such as oral contraceptives and GnRH agonists often recommended as first-line treatments. A meta-analysis found that hormonal treatments reduce pain scores by approximately 50%-70%; however, long-term use requires careful monitoring due to potential side effects like bone density loss [25]. The American College of Obstetricians and Gynecologists (ACOG) guidelines recommend hormonal therapy as an effective option for managing endometriosis-related pain [82].

Surgical interventions, particularly laparoscopy, are also widely used. The European Society of Human Reproduction and Embryology (ESHRE) guidelines suggest that laparoscopic surgery should be considered for patients with severe pain or those who do not achieve relief from medical treatments [83]. Future research directions should focus on several key areas to enhance treatment effectiveness and patient outcomes. One promising area is the integration and advancement of robotic-assisted surgery. Robotic-assisted laparoscopic surgery (RALS) has shown the potential to improve surgical precision and reduce recovery times [84]. A meta-analysis comparing RALS to conventional laparoscopy revealed no significant differences in intraoperative and postoperative complications. However, RALS was associated with longer operative durations and extended hospital stays. Despite these limitations, RALS provides enhanced precision, minimizes surgeon fatigue, and offers a more rapid learning curve [85].

The Da Vinci Surgical System, a leading robotic platform, has been instrumental in performing complex endometriosis surgeries with improved outcomes. Studies have reported that RALS can effectively manage deep infiltrating endometriosis, with a notable reduction in pain scores and improved quality of life [86]. Future research should also aim to address the high costs and extensive infrastructure requirements associated with robotic surgery. Developing cost-effective robotic systems and enhancing accessibility, particularly in smaller or rural hospitals, is crucial. Furthermore, advancements in tactile feedback technology could further improve the efficacy and safety of RALS [87]. Another important area for future research is the identification of biomarkers specific to endometriosis subtypes. This can help predict treatment responses and tailor therapies more effectively. Exploring the molecular mechanisms underlying endometriosis can lead to the development of targeted therapies with fewer side effects [88].

Future research should also focus on personalized treatment plans based on genetic, hormonal, and immunologic factors. Identifying biomarkers for endometriosis subtypes can help predict treatment response and improve patient outcomes. Additionally, exploring the molecular mechanisms underlying endometriosis can lead to the development of targeted therapies with fewer side effects.

## Conclusions

Hormonal therapies effectively reduce endometriosis-related pain by inducing a hypoestrogenic state, though they come with side effects, and symptoms often recur after discontinuation. Surgical interventions are also effective for pain management and improving fertility but carry varying risks and complications. Complementary therapies show promise for pain management with supportive evidence for their effectiveness and safety, although further research is needed. Non-pharmacological options like physical therapy, exercise, and psychological interventions offer safe, long-term pain management and improved quality of life.

Emphasizing patient preferences and quality of life is crucial, highlighting the need for personalized, multidisciplinary management plans that balance treatment effectiveness, cost-effectiveness, and minimal side effects. Future research should focus on robotic-assisted surgery, biomarker identification, and personalized treatment plans to enhance treatment effectiveness and patient outcomes.
